# Polymeric nanoparticle-mediated GBA1 gene therapy is neuroprotective in a preclinical model of Parkinson’s disease

**DOI:** 10.1007/s13346-025-01944-3

**Published:** 2025-08-20

**Authors:** Mohit Kwatra, Gijung Kwak, Haolin Li, Jung Soo Suk, Han Seok Ko

**Affiliations:** 1 Institute for Cell Engineering, School of Medicine, Johns Hopkins University, Baltimore, MD 21205, USA; 2 Department of Neurology, School of Medicine, Johns Hopkins University, Baltimore, MD 21205, USA; 3 Department of Neurosurgery, School of Medicine, University of Maryland, Baltimore, MD 21201, USA; 4 Medicine Institute for Neuroscience Discovery (UM-MIND), School of Medicine, University of Maryland, Baltimore, MD 21201, USA; 5 Department of Chemical and Biomolecular Engineering, School of Engineering, Johns Hopkins University, Baltimore, MD 21218, USA; 6 Department of Neurosurgery, School of Medicine, Johns Hopkins University, Baltimore, MD 21205, USA; 7 Present address: Department of Ophthalmology, College of Medicine, Medical University of South Carolina (MUSC), Charleston, SC 29425, USA

**Keywords:** Glucocerebrosidase 1, GCase, Αlpha-synuclein, Intracranial gene therapy, Convection-enhanced delivery, Polymeric nanoparticle, Neuroprotection, Neuroinflammation

## Abstract

Parkinson’s disease (PD) is a debilitating neurodegenerative disorder characterized by the progressive loss of dopaminergic neurons in the substantia nigra (SN). It manifests with hallmark motor symptoms such as tremors, rigidity, and bradykinesia, as well as severe non-motor complications. Current therapies provide symptomatic relief but fail to halt or reverse neurodegeneration, emphasizing that a disease-modifying treatment option is sorely needed. Mutations in *glucocerebrosidase 1 (GBA1)* gene encoding GCase or mutation-free reduction of GCase activity disrupt lysosomal function and drive α-synuclein (α-syn) accumulation, thereby leading to neuronal and motor function loss. To this end, restoring GCase activity by GBA1 gene therapy would potentially benefit a broad PD population with or without the genetic risk by intervening with the natural trajectory of the disease. In this study, we implemented localized GBA1 gene therapy by intracranial convection-enhanced delivery of plasmid DNA comprising human GBA1 gene carried by engineered polymeric nanoparticles capable of mediating widespread neuronal transgene expression. In an α-syn preformed fibril (PFF)-induced mouse model of PD, our therapeutic strategy mediated robust human GBA1 transgene expression in the SN to significantly reduce α-syn aggregation/accumulation, preserve tyrosine hydroxylase-positive dopaminergic neurons, and mitigate neuroinflammation. Remarkably, motor deficits were markedly improved, as demonstrated by grip strength, pole, and open field tests. These findings underscore the transformative potential of our nanoparticle-based GBA1 gene therapy in addressing the limitations of current standard-of-care treatments. We expect that our therapeutic strategy, upon clinical development and translation, may contribute to shifting the therapeutic paradigm from the current symptomatic management toward disease modification to ultimately provide PD patients with a curative therapeutic option.

## Introduction

Parkinson’s disease (PD) is a chronic neurodegenerative disease characterized by the accumulation of misfolded α-synuclein (α-syn) aggregates forming Lewy bodies, which disrupt cellular function and lead to the progressive loss of dopamine (DA) neurons in the substantia nigra (SN) [1, 2]. The standard of care for PD therapy, such as levodopa, a precursor of dopamine, remains symptomatic relief [3, 4]. Although motor symptoms are temporarily improved by the treatment, the disease continues to progress over time, making it hard to suppress the development of motor symptoms [5]. Extensive efforts to treat PD have focused on dopamine restoration, neuroprotection, and neuroregeneration [4, 6, 7]; however, interventions that only enhance dopamine production without preventing neurodegeneration have shown limited long-term success. Thus, the pathological progress of PD, such as lysosomal dysfunction, has emerged as a promising target for disease-modifying therapies (DMTs), yet their clinical translation remains challenging [8].

Recent evidence highlights lysosomal dysfunction as a central driver of PD pathogenesis, with mutations in the *glucocerebrosidase 1 (GBA1)* gene identified as one of the most significant genetic risk factors [9, 10]. GBA1 encodes glucocerebrosidase (GCase), a lysosomal enzyme essential for α-syn turnover [11]. Loss of GCase activity, whether due to GBA1 mutations or other pathological stressors, disrupts lysosomal function, impairs mitochondria and autophagy, and promotes the accumulation of misfolded proteins, including α-syn [9, 12–15]. This dysfunction triggers endoplasmic reticulum stress, neuroinflammation, and exacerbated neurodegeneration-hallmarks of PD progression [10, 13, 16–21]. Although GBA1 mutations are found in 7–15% of PD patients [22], reduced GCase activity is also observed in more prevalent idiopathic cases [23, 24], making GBA1 gene therapy a broadly applicable strategy. Unlike symptomatic treatments that merely replenish dopamine, GBA1 gene therapy aims to restore lysosomal function, enhance protein clearance, and mitigate neurodegeneration. Several preclinical studies using adeno-associated virus (AAV)-based GBA1 gene therapy have demonstrated neuroprotection and functional improvements [25–28]. However, viral vectors often lead to challenges such as therapy-inactivating immunogenicity, pro-inflammatory response, and/or potential off-target genomic integration [29]. In addition, current virus-based gene therapy products approved for treating rare genetic disorders cost $0.85–3.8M [30, 31], and thus a broad implementation of such an approach on more prevalent diseases, such as PD, may not be feasible. These limitations underscore the need for cost-affordable virus-free gene delivery platforms that enable efficient GCase expression and activity while minimizing safety risks.

We previously showed that non-adhesive polyethylene glycol-coated poly(β-amino ester)-based nanoparticles (PEG-PBAE NPs) efficiently penetrated and widely dispersed in the brain due to the reduced adhesive interactions with brain extracellular matrix (ECM) and enhanced physiologically stability [32, 33]. In addition, hydrolytic cleavage of the ester bonds on the backbone of the PBAE polymers facilitates intracellular release of nucleic acid payloads from PBAE-based NPs [34–37]. More recently, we also demonstrated that PEG-PBAE NPs were capable of transfecting key brain parenchymal cells, including neurons and astrocytes, in vivo [38]. Further, PEG-PBAE NPs exhibit excellent safety, low immunogenicity, and affordable manufacturing cost compared to AAVs, and thus are amenable to repeated treatments to enable sustained therapeutic transgene expression [39, 40]. To this end, we engineered PEG-PBAE NPs carrying plasmid DNA (pDNA) encoding human GBA1 gene (PEG-PBAE/pGBA1) and investigated the therapeutic potential as a DMT for PD in an α-syn preformed fibril (PFF)-induced mouse model of PD following intracranial convection-enhanced delivery (CED). Of note, CED, by creating a pressure gradient, facilitates the dispersion of non-adhesive NPs in the brain [41]. The performance of our therapeutic strategy was evaluated by comprehensive assessments of GBA1 transgene expression, α-syn pathology, neuroprotection, neurobehavior functions, and neuroinflammation.

## Materials and methods

### Polymer synthesis

PBAE was synthesized via Michael addition reactions, as previously described [33]. Briefly, 1,4-butanediol diacrylate (Millipore Sigma, Burlington, MA, USA) and 4-amino-1-butanol (Millipore Sigma) were polymerized at a molar ratio of 1.05:1 or 1.1:1 for 20 h at 90 °C. PBAE was purified with precipitation in cold diethyl ether (DE) (Thermo Fisher Scientific, Waltham, MA, USA) and dried in a vacuum for 3 days. To modify PBAE with acrylate functional groups, 500 mg of PBAE dissolved in dichloromethane (DCM) (Thermo Fisher Scientific) at 100 mg/mL was further reacted with 1,4-butanediol diacrylate at a quarter molar equivalent of the amount used for the initial PBAE polymerization reaction for 16 h at room temperature (RT). After cold DE-based precipitation and vacuum drying, number average molecular weight (MW) and polydispersity index of acrylated PBAE polymerized at a molar ratio of 1.05:1 or 1.1:1 were measured as 6.3 kDa and 1.37 or 4.1 kDa and 1.31, respectively, by gel permeation chromatography. To cap the terminal acrylate ends of PBAE, 200 mg of the high-MW (~ 6 kDa) and the low-MW (~ 4 kDa) PBAE dissolved in DCM at 100 mg/mL were reacted with 30 molar equivalents of 1,11-diamino-3,6,9-trioxaundecane (Millipore Sigma) and 1,2-diaminoethane (Millipore Sigma), respectively, for 16 h at RT. After cold DE-based precipitation and vacuum drying, 200 mg of 4 kDa PBAE end-capped with 1,2-diaminoethane dissolved in DCM at 100 mg/mL was reacted with 2.2 molar equivalents of 5 kDa methoxy PEG-epoxide (Creative PEGWorks, Durham, NC, USA) for 16 h at RT. The resultants were extensively dialyzed in a regenerated cellulose membrane tube having a 10 kDa MW cut-off (MWCO) for 3 days at 4 °C against methanol (Thermo Fisher Scientific) and PEG-PBAE was purified with cold DE-based precipitation and vacuum drying. The chemical structures of PBAE and PEG-PBAE are shown in Figure S1. PBAE and PEG-PBAE were stored at −80 °C and dissolved in anhydrous dimethyl sulfoxide at 100 mg/mL to be used for NP formulation.

### pDNA preparation

Bacterial cells transformed with pDNA expressing human GCase (hGCase) followed by T2A linker and ZsGreen1 (ZG1) driven by cytomegalovirus (CMV) promoter, pGBA1, were purchased from VectorBuilder (Chicago, IL, USA) (Figure S2). pDNA expressing luciferase driven by CMV promoter, pLuc, was a kind gift from Professor Alexander M. Klibanov (MIT). Competent E. coli DH5α bacterial cells (New England Biolabs, Ipswich, MA, USA) were transformed with pLuc. Bacterial cells transformed with pGBA1 or pLuc were placed on an agarose plate containing 100 μg/mL ampicillin (Millipore Sigma) or 50 μg/mL kanamycin (Millipore Sigma), respectively, and incubated at 37 °C in a humid chamber. After overnight incubation, a single colony of bacterial cells for pGBA1 or pLuc was expanded in 500 mL of sterilized Lennox Broth (LB) media (Millipore Sigma) containing 100 μg/mL ampicillin or 50 μg/mL kanamycin, respectively. When the optical density at 600 nm wavelength of the bacteria culture fell in a range of 3–3.5 as measured by a microplate reader (BioTek, Winooski, VT, USA), bacteria cells were lysed and pGBA1 or pLuc was purified using EndoFree Plasmid Mega Kit (Qiagen, Hilden, Germany) as per manufacturer’s protocol. The final pDNA pellet was resuspended in DNase/RNase-free distilled water (Thermo Fisher Scientific) at 1 mg/mL and stored at −80 °C until use.

### PEG-PBAE NP formulation

PEG-PBAE NPs were prepared by a formulation method previously reported [33, 38]. A blend of PBAE end-capped with 1,11-diamino-3,6,9-trioxaundecane and PEG-PBAE was prepared at a PBAE weight ratio of 3:2. PEG-PBAE NPs were formulated by vigorous mixing of polymer solution and pDNA solution at a volumetric ratio of 1:5 and PBAE-to-pDNA weight ratio at 60:1. After 10 min of incubation for NP assembly at RT, the solution was concentrated with centrifugation at 1,000 × g for 15 min at 4 °C in 100 kDa MWCO Amicon Ultra Centrifugal Filters (Millipore Sigma). As a washing process, the concentrated PEG-PBAE NP solution was diluted 10 times with DNase/RNase-free distilled water (Thermo Fisher Scientific) and re-centrifuged at 1,000 × g for 15 min at 4 °C to remove residual polymers. After three washes, PEG-PBAE NP solution was concentrated to 1 mg/mL of pDNA concentration in normal saline for subsequent experiments.

### Agarose gel electrophoresis

pGBA1 or PEG-PBAE/pGBA1 equivalent to 300 ng of pDNA were loaded into the wells of a 1% agarose gel made with SYBR Safe DNA Gel Stain (Thermo Fisher Scientific). Electrophoresis was conducted at 100 V for 30 min in Tris-acetate-EDTA buffer. The gel was imaged using a ChemiDoc imaging system (Bio-RAD, Hercules, CA, USA).

### α-syn PFF preparation

Competent E. coli BL21 bacterial cells (Lucigen Corporation, Middleton, WI, USA) transformed with pRK172 vector encoding a full-length mouse α-syn were expanded in LB media containing 100 μg/mL ampicillin, as described above. Mouse α-syn was purified, as previously described [42]. To remove bacterial endotoxins, α-syn was further purified with Toxineraser Endotoxin Removal Kit (GenScript Biotech, Piscataway, NJ, USA). Endotoxin level was confirmed to be < 0.5 EU/mL using ToxinSensor Chromogenic LAL Endotoxin Assay Kit (GenScript Biotech). Purified α-syn was stored at − 80 °C until fibrillization. Before use, aliquots were centrifuged at 12,000 × g for 15 min at 4 °C to remove aggregates. The supernatant containing α-syn monomers was collected and quantified by BCA Protein Assay Kit (Pierce, Rockford, IL, USA). To induce α-syn fibril formation, 5 mg/mL α-syn was incubated at 37 °C with shaking for 5–7 days. The resulting fibrils were sonicated at 20% amplitude with 60 pulses (0.5-second on/off cycles) by Branson SFX150 Sonifier (Marshall Scientific, Hampton, NH, USA) and utilized for striatal CED to induce mouse models of PD.

### Transmission electron microscopy (TEM)

PEG-PBAE NPs carrying pLuc (PEG-PBAE/pLuc) equivalent to 1 μg of pDNA were loaded on a carbon type-B copper grid (Ted Pella, Redding, CA, USA) and dried for 6 h. After rinsing with deionized water for 1 min, the sample was stained with UranylLess EM Stain (Electron Microscopy Sciences, Hatfield, PA, USA) for 1 min. The excess staining solution was gently removed with filter paper, and the grids were air-dried. Imaging was performed with a Hitachi H7600 TEM (Hitachi High-Technologies, Tokyo, Japan).

Five hundred nanograms of pre- or post-sonicated α-syn PFF were loaded on a carbon type-B copper grid (Ted Pella) for 5 min. After rinsing with 50 mM Tris-HCl buffer (pH 7.4) for 1 min three times, the sample was stained with 2% uranyl formate (Electron Microscopy Sciences) for 1 min. The excess staining solution was gently removed with filter paper, and the grids were air-dried. Imaging was performed with a Phillips CM 120 TEM (Philips Lighting Holding B.V., Eindhoven, Netherlands).

### Dynamic light scattering (DLS)

PEG-PBAE/pLuc or PEG-PBAE/pGBA1 was diluted to a pDNA concentration of 5 μg/mL in 10 mM NaCl at pH 7.4. The diluted NP solution was transferred into a UV-transparent cuvette or a Capillary Zeta cell (Malvern Instruments, Malvern, UK) and used to measure hydrodynamic diameters or ζ-potentials, respectively, using a Zetasizer Nano ZS (Malvern Instruments).

### Intracranial CED of PEG-PBAE/pGBA1 to SN

Male C57Bl/6j mice (9–10-week-old) were purchased from Jackson Laboratory (Bar Harbor, ME, USA). Mice were handled in accordance with the guidelines and policies of the Institutional Animal Care and Use Committee at Johns Hopkins University or University of Maryland Baltimore. Mice were anesthetized by intraperitoneal injection of a 0.1 mL mixture of Ketamine (100 mg/kg) and Xylazine (10 mg/kg). The heads of the animals were shaved with a hair trimmer. A midline scalp incision was made to expose the coronal and sagittal sutures. The injection coordinates were carefully defined as + 3.2 mm anteroposterior (AP), + 1.2 mm mediolateral (ML), and − 4.6 mm dorsoventral (DV) to target SN. 2 μL of saline, pGBA1, PEG-PBAE/pLuc, PEG-PBAE/pGBA1, or Cy5-labeled PEG-PBAE/pGBA1 equivalent to 1- or 2-μg pDNA was infused with a 33-gauge Neuros syringe (Hamilton, Reno, NV, USA) or Nanofil gas tight syringe (World Precision Instruments, Sarasota, FL, USA) at a rate of 0.2 μL/min as controlled by a Chemyx Nanojet Injector Module (Chemyx, Stafford, TX, USA) or UMP3 micro syringe pump injector (World Precision Instruments). When the infusion was completed, the needle was left in place for 5 min and carefully withdrawn at 1 mm/min. Post-surgery, the mice received 0.5 mL of subcutaneous normal saline for hydration and 100 μL of RIMADYL (carprofen) injectable (5 mg/kg) (ParsippanyTroy Hills, NJ, USA) or 400 μL of Buprenorphine HCl (0.5 mg/kg) (Covetrus, Dublin, OH, USA). The surgical site was closed with sterile absorbable sutures. Each mouse was monitored regularly during recovery to ensure proper healing and well-being.

### Biodistribution of PEG-PBAE NPs

After 30-minute post-intracranial CED of saline or Cy5labeled PEG-PBAE/pGBA1 equivalent to 2 μg pDNA, the major organs, including brain, heart, liver, kidney, lung, and spleen, were harvested and imaged using an in vivo imaging system (IVIS, Perkin Elmer, Waltham, MA, USA) at the excitation/emission wavelength of 640/680 nm. For image-based quantification, the fluorescence intensity of each organ was measured with Living Image software (Perkin Elmer).

### Safety assessment

Healthy animals were treated with intracranial CED of saline or PEG-PBAE/pGBA1 equivalent to 2 μg pDNA. After 2 days, the blood samples were collected with Mini-Collect CAT Serum Sep Tubes (Greiner Bio-One, Monreo, NC, USA), gently shaken by inverting the tubes 8–10 times, and incubated for 10 min at RT. The blood samples were centrifuged at 3,000 × g for 10 min at 18 °C, and the supernatant, serum sample, was collected. To conduct blood chemistry for the liver or kidney metabolic function indicators, the undiluted serum samples were analyzed with Short-Tox panel including alkaline phosphatase (ALP), aspartate transaminase (AST), alanine transaminase (ALT), and blood urea nitrogen (BUN), by IDEXX Laboratories (Westbrook, ME, USA). The total IgG concentration in serum samples was measured with Easy-Titer Mouse IgG Assay Kit (Thermo Fisher Scientific) according to the manufacturer’s instructions. Briefly, 20 μL of the mouse IgG-sensitized beads were incubated with 1,000 times diluted serum samples for 5 min at RT. 100 μL of blocking buffer was added and incubated for another 5 min at RT. The absorbance of the samples was measured by a microplate reader (BioTek) at 450 nm.

### PD animal model establishment and PEG-PBAE/pGBA1 treatments

Male C57BL/6j mice (6–10 weeks old) received intracranial saline or α-syn PFF administration with surgical procedures described above. The injection coordinates were carefully defined as + 0.5 mm AP, + 2.0 mm ML, and – 3.0 mm DV to target the dorsal striatum. 2 μL of saline or α-syn PFF equivalent to 5 μg α-syn PFF was infused at a rate of 0.2 μL/min. After 2 months of α-syn accumulation and pathological development of PD, the mice received saline or PEG-PBAE/pGBA1 equivalent to 2 μg pDNA via SN-targeted CED once a week up to 4 doses. The brain harvesting and neurobehavior tests were conducted at the 3-month post-striatal saline or α-syn PFF administration.

### Immunofluorescence (IF) and immunohistochemistry (IHC)

For brain harvesting, transcardiac perfusion was performed with phosphate-buffered saline (PBS) and followed by 4% paraformaldehyde (PFA). The brains were carefully excised and further fixed in 4% PFA for 24 h at RT. After fixation, they were transferred to a 30% sucrose solution in PBS and stored at 4 °C. Floating coronal brain sections were obtained using an HM 450 Sliding Microtome (Thermo Fisher Scientific) with a 40 μm thickness. The sections were washed three times with PBS and blocked in PBS containing 0.2% Triton X-100 (TX) and 4% goat or donkey serum for 1 h at RT. After blocking, sections were washed three times with PBS-T (PBS containing 0.1% Tween 20) for 5 min each and utilized for IF and IHC.

For IF, the sections were incubated overnight at 4 °C with primary antibodies: anti-α-syn phosphorylated at serine 129 (pS129 α-syn) antibody (Cat #: 825701, Biolegend, San Diego, CA, USA), anti-tyrosine hydroxylase (TH) antibody (Cat #: NB300–109, Novus Biologicals, Littleton, CO, USA), anti-ionized calcium binding adaptor molecule 1 (IBA1) antibody (Cat #: 019–19741, FUJIFILM Cellular Dynamics, Madison, WI, USA), or anti-glial fibrillary acidic protein (GFAP) antibody (Cat #: 3670 S, Cell Signaling Technology, Danvers, MA, USA). After three PBS-T washes, sections were incubated with the appropriate secondary antibodies: Alexa Fluor 488-conjugated anti-goat antibody (Cat #: A-11008, Thermo Fisher Scientific) or Alexa Fluor 555-conjugated anti-rabbit antibody (Cat #: A-21422, Thermo Fisher Scientific) for 1 h at RT. After three PBS-T washes, sections were counterstained with DAPI (Thermo Fisher Scientific) for 15 min. Sections were then mounted on gelatin-coated slides (FD Neuro Technologies, Columbia, MD, USA) and allowed to dry overnight. After three PBS-T washes, the slides were mounted with Fluoromount-G Mounting Medium (Thermo Fisher Scientific). The sections were imaged with an A1 confocal laser microscope (Nikon, Tokyo, Japan) and analyzed by ImageJ software (NIH, Bethesda, MD, USA) [43, 44].

For IHC, the sections were incubated overnight at RT with primary antibodies: anti-pS129 α-syn antibody (Cat #: ab51253, Abcam, Cambridge, UK) or anti-TH antibody (Cat #: NB300–109, Novus Biologicals). After three PBS-T washes, sections were incubated for 2 h at RT with a biotinylated goat anti-rabbit secondary antibody (Vectastain Elite ABC Kit, Vector Laboratories, Burlingame, CA, USA). After three PBS-T washes, the slides were stained with Vectastain Elite ABC Kit (Vector Laboratories) or SigmaFast Diaminobenzidine (DAB) Peroxidase Substrate (Millipore Sigma). After immunostaining, sections were dehydrated and mounted with D.P.X. neutral mounting medium (Millipore Sigma). To enhance cytoarchitectural visualization for TH staining, sections were counterstained with 0.1% cresyl violet (Abcam) [45, 46]. The sections were imaged with an Axiophot photomicroscope (Carl Zeiss, Stuttgart, Germany) and analyzed by Stereo Investigator software (MBF Bioscience, Williston, VT, USA) that allowed for systematic and unbiased quantification of IHC staining across defined brain regions (i.e., SN and striatum).

### Western blot (WB) analysis

The ventral midbrains (VMBs) or hemispheres harvested from the treated brains were homogenized in lysis buffer containing 10 mM Tris-HCl, 150 mM NaCl, 5 mM EDTA, 1% TX, phosphatase inhibitor cocktail II and III (Millipore Sigma), and complete protease inhibitor mixture (Millipore Sigma). The lysates were centrifuged at 12,000 × g for 30 min at 4 °C and the supernatant was collected as TX-soluble fraction. The insoluble pellets were washed once with lysis buffer and further lysed with protein-denaturing lysis buffer, which is the lysis buffer added with 2% sodium dodecyl sulfate (SDS) and 0.5% sodium deoxycholate. The pellets were incubated for 30 min at 4 °C and subsequently sonicated at 30% amplitude with 5 pulses (5-second on/off cycles) by a Branson SFX150 Sonifier (Marshall Scientific). The lysates with protein-denaturing lysis buffer were centrifuged at 12,000 × g for 30 min at 4 °C, and the supernatants were collected as TX-insoluble fraction. The protein concentration of TX-soluble and TX-insoluble fractions was quantified by BCA Protein Assay Kit (Pierce). Samples were prepared in Laemmli Buffer (Bio-RAD) supplemented with 2-mercaptoethanol (Bio-RAD) and annealed at 100 °C for 5 min. The samples comprising 20 μg of protein content were subjected to electrophoresis with a 10–20% SDS-polyacrylamide gel (Thermo Fisher Scientific) and transferred onto a polyvinylidene fluoride membrane (Bio-RAD). The membranes were then blocked at RT in Tris-buffered saline (TBS)-T solution (pH 7.4, 20 mM Tris, 150 mM NaCl, and 0.1% Tween 20) supplemented with 5% bovine serum albumin. After an hour, the membranes with TX-soluble fractions were incubated overnight at 4 °C with primary antibodies: anti-hGCase antibody (Cat #: ab55080, Abcam), anti-TH antibody (Cat #: NB300–109, Novus Biologicals), anti-IBA1 antibody (Cat #: 016–20001, FUJIFILM), anti-GFAP antibody (Cat #: 3670 S, Cell Signaling Technology), anti-nuclear factor kappa B (NF-κB) p65 phosphorylated at serine 536 (pNF-κB p65) antibody (Cat #: 3033T, Cell Signaling Technology), anti-interleukin-1 beta (IL-1β) antibody (Cat #: 12242 S, Cell Signaling Technology), or anti-β-actin antibody (Cat #: 4967 S, Cell Signaling Technology). The membranes with TX-insoluble fractions were incubated overnight at 4 °C with primary antibodies: antipS129 α-syn antibody (Cat #: ab51253, Abcam) or anti-β-actin antibody (Cat #: 4967 S, Cell Signaling Technology). After three TBS-T washes, the membranes were incubated with secondary anti-mouse or rabbit IgG antibody conjugated with horseradish peroxidase (Cat #: 31430 or 31460, respectively, Thermo Fisher Scientific) for an hour at RT. After three TBS-T washes, protein bands were detected with the SuperSignal^™^ West Femto Maximum Sensitivity Substrate (Thermo Fisher Scientific) by an Amersham Imager 600 (GE Healthcare, USA). Densitometric analysis of protein bands was performed using ImageJ software (NIH) as previously described [43].

### Neurobehavioral test: grip strength test, pole test, and open field test

Neurobehavioral tests were conducted to evaluate motor and non-motor deficits in a blind manner to ensure unbiased results. All behavioral evaluations were conducted between 10:00 and 16:00 during the lights-on cycle, and the sessions were recorded for analysis. Before testing, mice were acclimated to the behavioral testing room for 30 min.

In grip strength test, neuromuscular strength was evaluated by measuring the maximum holding force exerted by the mice using a specialized force transducer system (Bioseb, Pinellas Park, FL, USA). The treated mice were allowed to grasp a metal grid using their forelimbs only or fore and hindlimbs together, depending on the test configuration. To measure the holding force, gentle traction was applied to the tail until the mouse released its grip on the grid. The transducer recorded the peak holding strength at the moment of release, providing a reliable and precise assessment of neuromuscular function [47–50].

In pole test, a pole, a 7.5 cm metal rod with a 9 mm diameter, wrapped in bandage gauze to provide grip. The treated mice were placed on the top of the pole, facing upwards, and the time taken to descend to the base of the pole was recorded. Before the actual testing, the mice were tested three times a day for 3 days to become accustomed to the task. On the test day, video recordings captured mouse performance during three separate pole test sessions and were utilized to analyze descending time, the number of paused trials, and head turn time [44, 47, 51].

In open field test, a rectangular plastic box measuring 40 × 40 × 40 cm^3^, which was divided into 36 equal sectors (6 × 6 grids; each square measuring 6.6 × 6.6 cm), was used as a test field. Each mouse was placed at the center of the field and allowed to explore freely for 5 min under dim lighting to minimize stress. Between the trials, the apparatus was thoroughly cleaned with 70% ethanol to eliminate residual odors. A video tracking system (ANY-maze, Wood Dale, IL, USA) was used to record and analyze locomotor activity [47, 49, 51–53].

### Statistical analysis

Data were analyzed using Prism version 6.0 (GraphPad Software, San Diego, CA, USA). Results are expressed as mean ± SEM. Statistical significance was determined using two-tailed unpaired t-test or one- or two-way ANOVA followed by appropriate post-hoc tests, with *p* < 0.05 considered significant.

## Results

### PEG-PBAE/pGBA1 enables widespread hGCase expression in the SN of healthy mouse brains

**Fig. 1:**
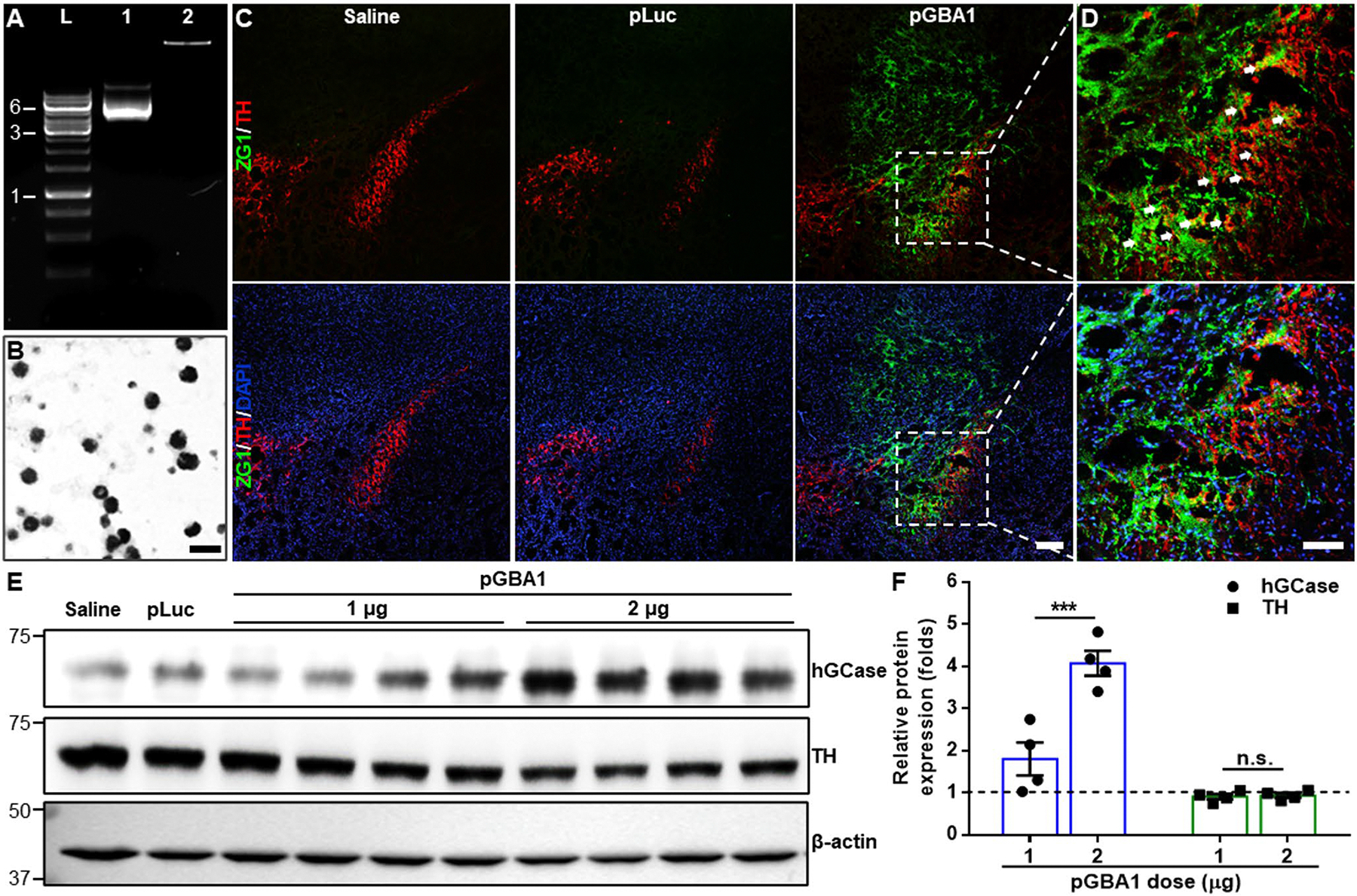
PEG-PBAE/pGBA1 provides overexpression of hGCase in TH-positive DA neurons in healthy mice following SN-targeted CED. (**A**) Representative agarose gel image showing robust packaging of pGBA1 in PEG-PBAE NPs. L: DNA ladder; lane 1: pGBA1; lane 2: PEG-PBAE/pGBA1. Sizes of pDNA in kb based on DNA ladder are indicated on the left side of gel image. (**B**) Representative transmission electron micrographs of PEG-PBAE/pLuc. Scale bar = 100 nm. (**C**) Representative confocal images of the SN area received saline, PEG-PBAE/pLuc, or PEG-PBAE/pGBA1 (2 μg pDNA). pGBA1 includes a reporter ZG1 encoding sequence. Scale bar = 200 μm. (**D**) High-magnified images of the white dot-lined square in C. Green: ZG1; red: TH; blue: nucleus. Areas where ZG1 and TH are co-located are indicated by white arrows. Scale bar = 100 μm. (**E**) Representative WB images showing expression of hGCase, TH, and β-actin in brain hemispheres received saline, PEG-PBAE/pLuc, or PEG-PBAE/pGBA1 (1 or 2 μg pDNA) via SN-targeted CED. Molecular weights in kDa based on protein ladder are indicated on the left side of blot images. (**F**) Quantification of relative hGCase and TH expression normalized by β-actin expression of PEG-PBAE/pGBA1 treated groups compared to saline group, based on the WB data in E. n.s.: no significance, ***p < 0.001 (two-tailed unpaired t-test)

PEG-PBAE NPs were utilized to compact pGBA1 at a PBAE-to-pDNA weight ratio of 60:1. Gel electrophoresis migration assay revealed that pGBA1 was fully compacted in PEG-PBAE NPs (Fig. 1A). TEM image showed the spherical morphology of PEG-PBAE NPs with < 100 nm geometric diameters (Fig. 1B). PEG-PBAE/pLuc and PEG-PBAE/pGBA1 exhibited hydrodynamic diameters of 65.2 ± 1.2 and 67.5 ± 1.6 nm and ζ-potentials of 2.4 ± 0.1 and 2.1 ± 0.2 mV, respectively (Table 1). To assess the ability of PEG-PBAE NPs to mediate transgene expression in vivo, saline, PEG-PBAE/pLuc, or PEG-PBAE/pGBA1 at a pDNA dose of 2 μg was intracranially administrated to the SN of healthy mice via CED. After 48 h post-administration, ZG1 reporter expressed from pGBA1 was widely distributed in SN area in PEG-PBAE/pGBA1-treated brains, but not in saline- or PEG-PBAE/pLuc-treated brains (Fig. 1C). Of note, in PEG-PBAE/pGBA1-treated brain, ZG1 expression was colocalized with TH-positive DA neurons (Fig. 1D). Of note, ZG1 signal was not observed in the SN of mice treated with carrier-free pGBA1, similar to the observation with saline-treated mice (Figure S3). Also, we conducted WB analysis to confirm hGCase expression in SN. VMBs were harvested from the brains treated with saline, PEG-PBAE/pLuc, or PEG-PBAE/pGBA1 after 48 h post-administration. hGCase was overexpressed in PEG-PBAE/pGBA1 group, but not in saline and PEG-PBAE/pLuc groups (Figure S4A). The PEG-PBAE/pGBA1 group showed over an order of magnitude greater hGCase expression compared to the saline group in image-based quantification (Figure S4B). We also confirmed that hGCase expression by PEG-PBAE/pGBA1 was dose-dependent and the treatment did not affect the level of TH expression (Fig. 1E and F).

We next investigated whether locally administered PEG-PBAE/pGBA1 stayed in the brain or were systemically distributed. Intracranially administered Cy5-labeled PEG-PBAE/pGBA1 was fully retained in the brain and not found in other peripheral organs (Figure S5). In addition, serum was collected 48 h after administration of PEG-PBAE/pGBA1 via SN-targeted CED in healthy animals to quantify the concentrations of metabolic function indicators and total IgG. The concentrations of the indicators for liver, including ALP, AST, and ALT, and for kidney, including BUN, were not significantly altered in PEG-PBAE/pGBA1-treated animals compared to saline-treated animals (Figure S6). Furthermore, total mouse IgG concentrations in the serum of PEG-PBAE/pGBA1-treated animals were identical to those of saline-treated animals (Figure S7), indicating the absence of a humoral immune response.

### hGCase is overexpressed in the SN via PEG-PBAE/pGBA1 in α-syn PFF-induced mouse model of PD

Prior to use, quality control testing of α-syn PFF was performed on both pre-sonicated and post-sonicated PFFs, as previously described [54, 55]. Fibril morphology and amyloid content in the α-syn fibril preparations were confirmed using TEM (Figure S8A). After sonication, fragmented α-syn PFF showed enhanced Thioflavin-T fluorescence intensity, indicating increased reactive surface and seeding capacity of α-syn PFF to accelerate α-syn aggregation in the brain [56, 57] (Figures S8B and S8C). Additionally, seeding activity was validated by assessing pS129 α-syn immunoreactivity (Figure S8D), a marker of Lewy body pathology, in primary cortical neurons treated with sonicated α-syn PFF [54, 55].

**Fig. 2:**
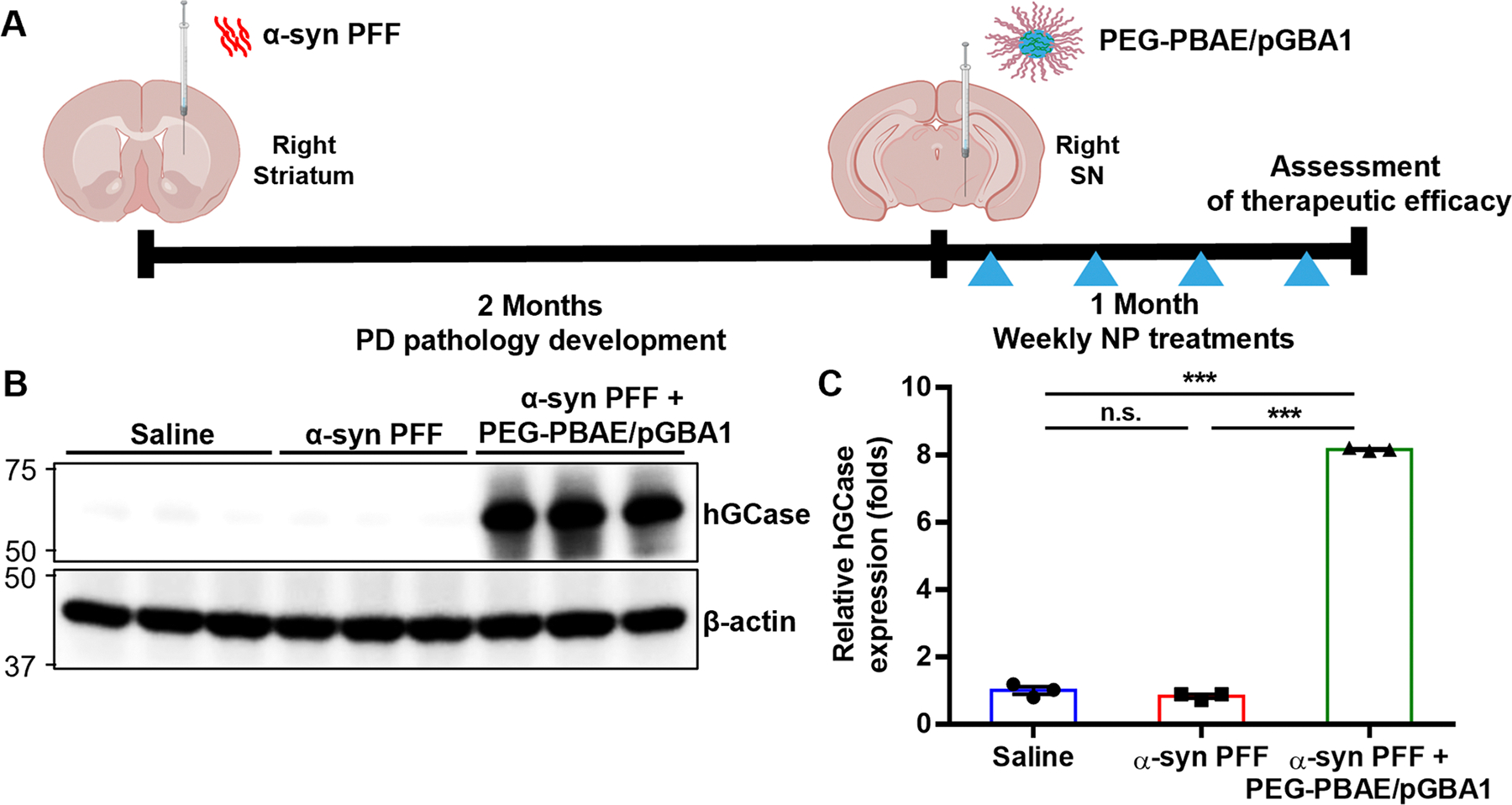
PEG-PBAE/pGBA1 mediates robust hGCase transgene expression in an α-syn PFF-induced mouse model of PD following SN-targeted CED. (**A**) Schematic timeline outlining the development of PD pathology and PEG-PBAE/pGBA1 treatments. Mice initially received saline or α-syn PFF (5 μg) once via striatum-targeted CED and followed 2 months later by saline or PEG-PBAE/pGBA1 (2 μg pDNA) once every week for 4 times via SN-targeted CED. Brains tissues were collected at the 3-month time point. (**B**) Representative WB images showing expression of hGCase and β-actin in the VMBs of mice received saline or α-syn PFF with or without PEG-PBAE/pGBA1 treatments, as described in the timeline in A. Molecular weights in kDa are indicated on the left side of blot images. (**C**) Quantification of relative hGCase expression normalized by β-actin expression, based on the WB data in B. n.s.: no significance, **p < 0.01, ***p < 0.001 (one-way ANOVA followed by Tukey’s post-hoc test)

A mouse model of PD was induced by sonicated α-syn PFF administration to striatum, as shown in Fig. 2A. Two months post-α-syn PFF injection, saline or PEG-PBAE/pGBA1 at a pDNA dose of 2 μg was intracranially administered into the SN once a week up to 4 doses. Three months after striatal saline or α-syn PFF administration (i.e., one month after the initiation of the treatment), the treated brains were harvested for neuropathological and neurochemical analysis. The α-syn PFF-induced PD mice treated with PEG-PBAE/pGBA1 (α-syn PFF + PEG-PBAE/pGBA1 group) were compared with healthy saline-treated mice and α-syn PFF-induced PD mice that received saline. Consistent with our observation with healthy brains (Fig. 1), hGCase expression was significantly increased in the α-syn PFF + PEG-PBAE/pGBA1 group, but not in the saline and α-syn PFF groups (Fig. 2B and C).

### Augmentation of hGCase via PEG-PBAE/pGBA1 reduces Lewy body pathology in the SN in α-syn PFF-induced mouse model of PD

**Fig. 3:**
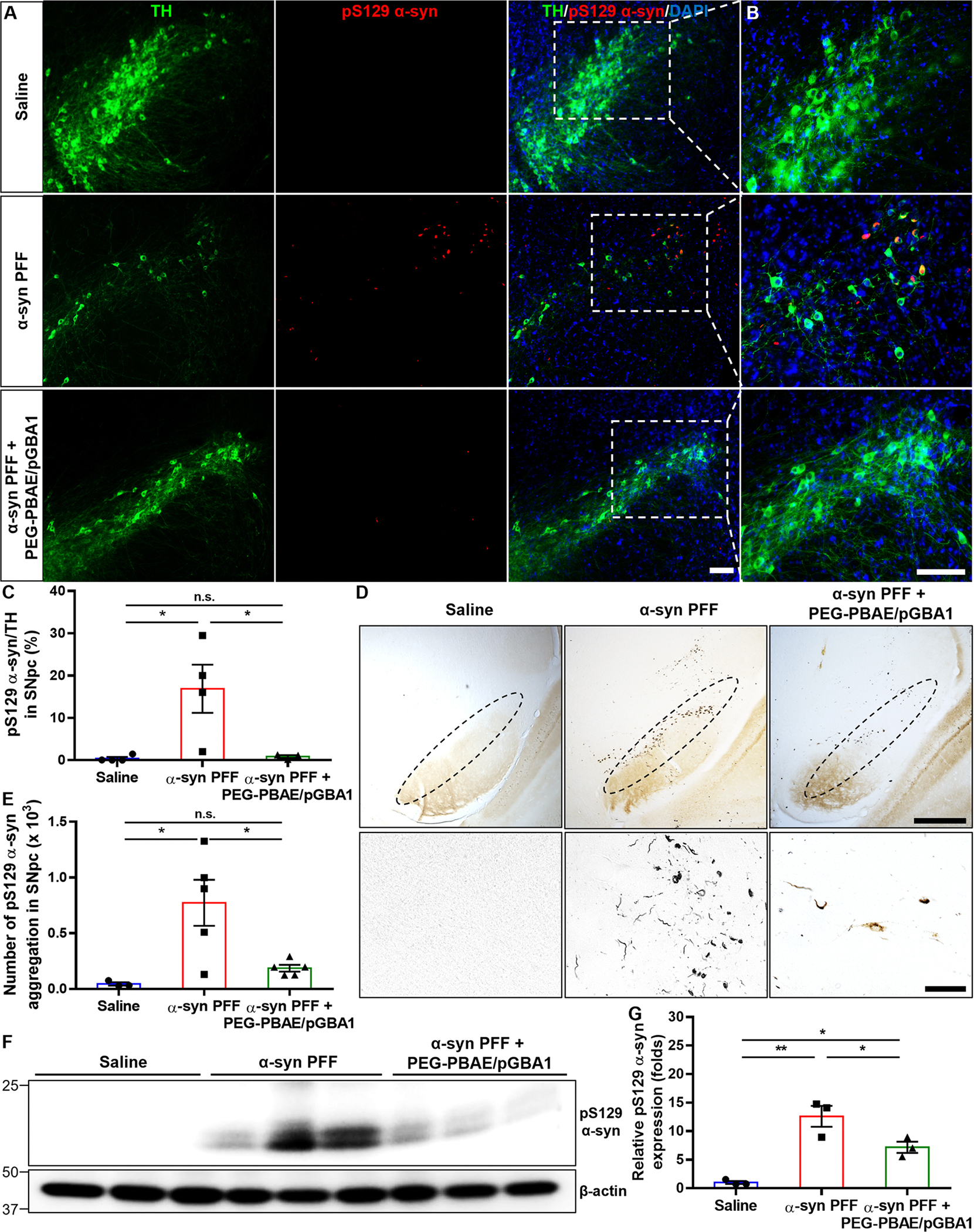
Overexpression of hGCase via SN-targeted CED of PEG-PBAE/pGBA1 decreases Lewy body pathology (pS129 α-syn immunoreactivity) in the SN in an α-syn PFF-induced mouse model of PD. (**A**) Representative confocal images of the SN in mice received saline or α-syn PFF with or without PEG-PBAE/pGBA1 treatments, as outlined in the timeline in Figure 2A. Scale bar = 100 μm. (**B**) High-magnified images of the white dot-lined square in A. Green: TH; red: pS129 α-syn; blue: nucleus. Scale bar = 100 μm. (**C**) Quantification of the percentage of pS129 α-syn-positive staining in TH-positive neurons in the SNpc, based on the panels in A and B. (**D**) Representative images of IHC staining for pS129 α-syn in the SNpc of mice received saline or α-syn PFF with or without PEG-PBAE/pGBA1 treatments, as indicated in the timeline in Figure 2A. The SNpc area is marked by the black dot-lined ellipse in the top panels. Scale bar in top/bottom panels = 200/50 μm. (**E**) Quantification of the number of pS129 α-syn aggregations in the SNpc, based on the panels in D. (**F**) Representative WB images showing the levels of pS129 α-syn and β-actin in TX-insoluble fractions obtained from the VMBs of mice received saline or α-syn PFF with or without PEG-PBAE/pGBA1 treatments, as described in the timeline in Figure 2A. Molecular weights in kDa are indicated on the left side of blot images. (**G**) Quantification of relative pS129 α-syn levels normalized by β-actin, based on the WB data in F. n.s.: no significance, *p < 0.05, **p < 0.01 (one-way ANOVA followed by Tukey’s post-hoc test)

Lewy body pathology was assessed in the treated brains. IF staining for TH and pS129 α-syn antibodies showed significant loss of TH-positive DA neurons and elevation of pS129 α-syn immunoreactivity, a marker of pathological α-syn [44], in the SN of α-syn PFF-injected mice (Fig. 3A). PEG-PBAE/pGBA1 treatments markedly reduced pS129 α-syn immunoreactivity in the SN (Fig. 3A), particularly in TH immunoreactive positive neurons (Fig. 3B).

Quantification showed the percentage of pS129 α-syn/TH in the substantia nigra pars compacta (SNpc) increased to 16.9 ± 2.9% in α-syn PFF mice but reduced to 0.9 ± 0.2% with PEG-PBAE/pGBA1 treatments, comparable to saline group (Fig. 3C). The number of pS129 α-syn positive aggregates in the SN of α-syn PFF group increased to a mean of 773 ± 92 but reduced to 186 ± 14 with PEG-PBAE/pGBA1 treatments, similar to the saline controls as assessed by DAB staining (Fig. 3D and E). In addition, WB analysis confirmed a 12.6 ± 1.1-fold increase in TX-insoluble pS129 α-syn levels in α-syn PFF mice, which was significantly reduced by PEG-PBAE/pGBA1 treatments (Fig. 3F and G).

### Overexpression of hGCase via PEG-PBAE/pGBA1 protects TH-positive DA neurons in SN and striatum in the α-syn PFF-induced mouse model of PD

**Fig. 4:**
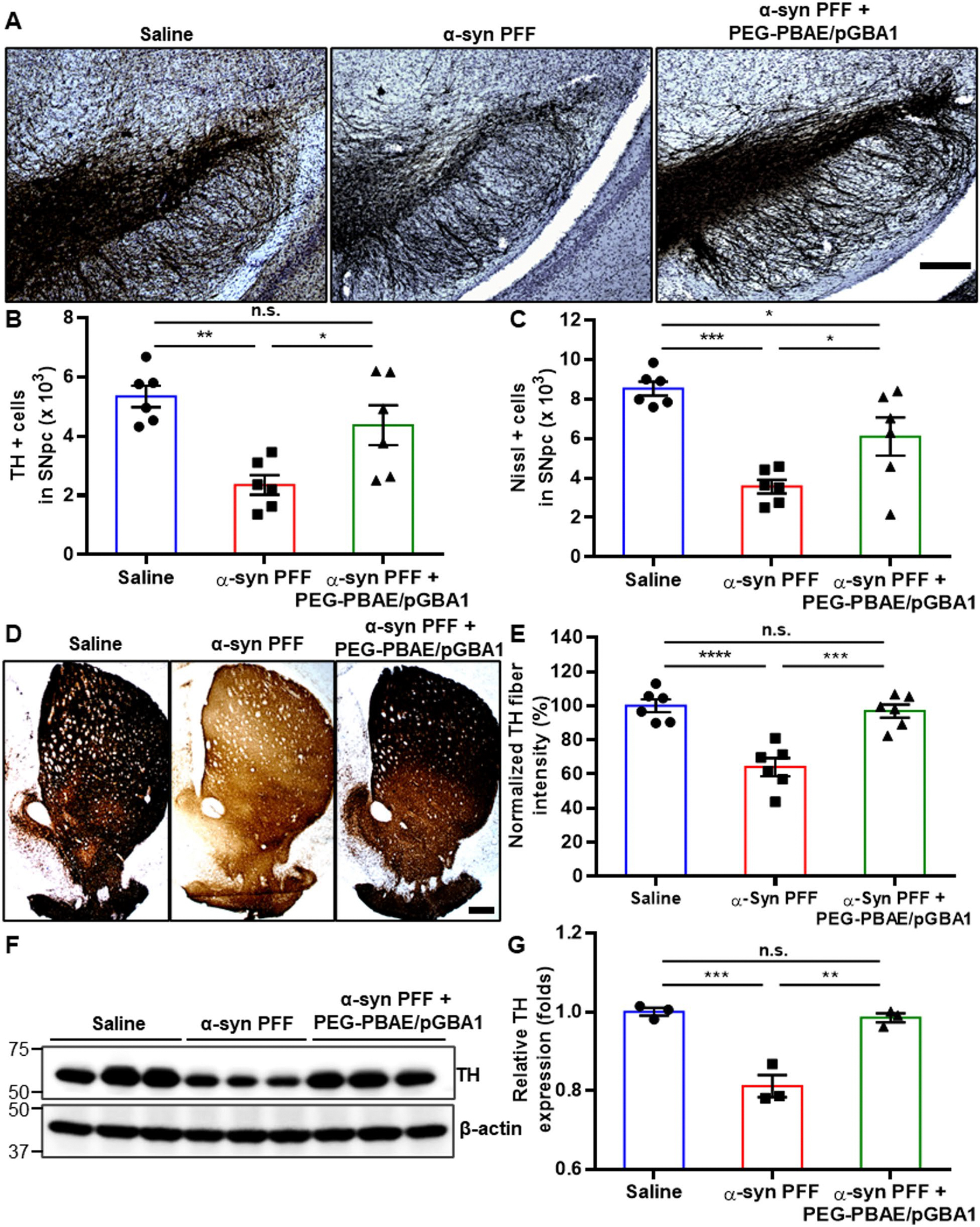
Overexpression of hGCase by SN-targeted CED of PEG-PBAE/pGBA1 rescues nigrostriatal TH-positive DA neurons in an α-syn PFF-induced mouse model of PD. (**A**) Representative images of IHC staining for TH in the SN from mice received saline or α-syn PFF with or without PEG-PBAE/pGBA1 treatments, as outlined in the timeline in Fig. 2A. Scale bar = 200 μm. Quantification of (**B**) TH-positive DA neurons and (**C**) Nissl-positive neurons in the SNpc. (**D**) Representative images of IHC staining for TH in the striatum from mice received saline or α-syn PFF with or without PEG-PBAE/pGBA1 treatments, as indicated in the timeline in Fig. 2A. Scale bar = 500 μm. (**E**) Quantification of relative TH fiber intensity in the striatum, based on image panels in D. (**F**) Representative WB images showing expression of TH and β-actin in the VMBs of mice received saline or α-syn PFF with or without PEG-PBAE/pGBA1 treatments, as described in the timeline in Fig. 2A. Molecular weights in kDa are indicated on the left side of blot images. (**G**) Quantification of relative TH levels normalized by β-actin expression, based on the WB data in F. n.s.: no significance, *p < 0.05, **p < 0.01, ***p < 0.001 (one-way ANOVA followed by Tukey’s post-hoc test)

We examined TH-positive DA neurons in the SN and striatum by IHC. Consistent with TH staining images in Fig. 3A, quantification revealed a 44.0 ± 2.5% reduction in TH-positive neurons in the SNpc of α-syn PFF group compared to the saline group, whereas PEG-PBAE/pGBA1 treatments restored the number of TH-positive neurons (Fig. 4A and B). Similarly, Nissl-positive cells in the SNpc were decreased by 41.8 ± 1.6% in α-syn PFF group, which was restored by PEG-PBAE/pGBA1 treatments (Fig. 4C). IHC analysis of the striatum revealed a significant reduction in TH fiber density in α-syn PFF group, but the level was normalized by PEG-PBAE/pGBA1 treatments (Fig. 4D and E). Likewise, WB analysis confirmed a significant reduction in TH levels in α-syn PFF group, which was restored to the normal level by PEG-PBAE/pGBA1 treatments (Fig. 4F and G).

### PEG-PBAE/pGBA1 treatments restore neurobehavior deficits in the α-syn PFF-induced mouse model of PD

**Fig. 5:**
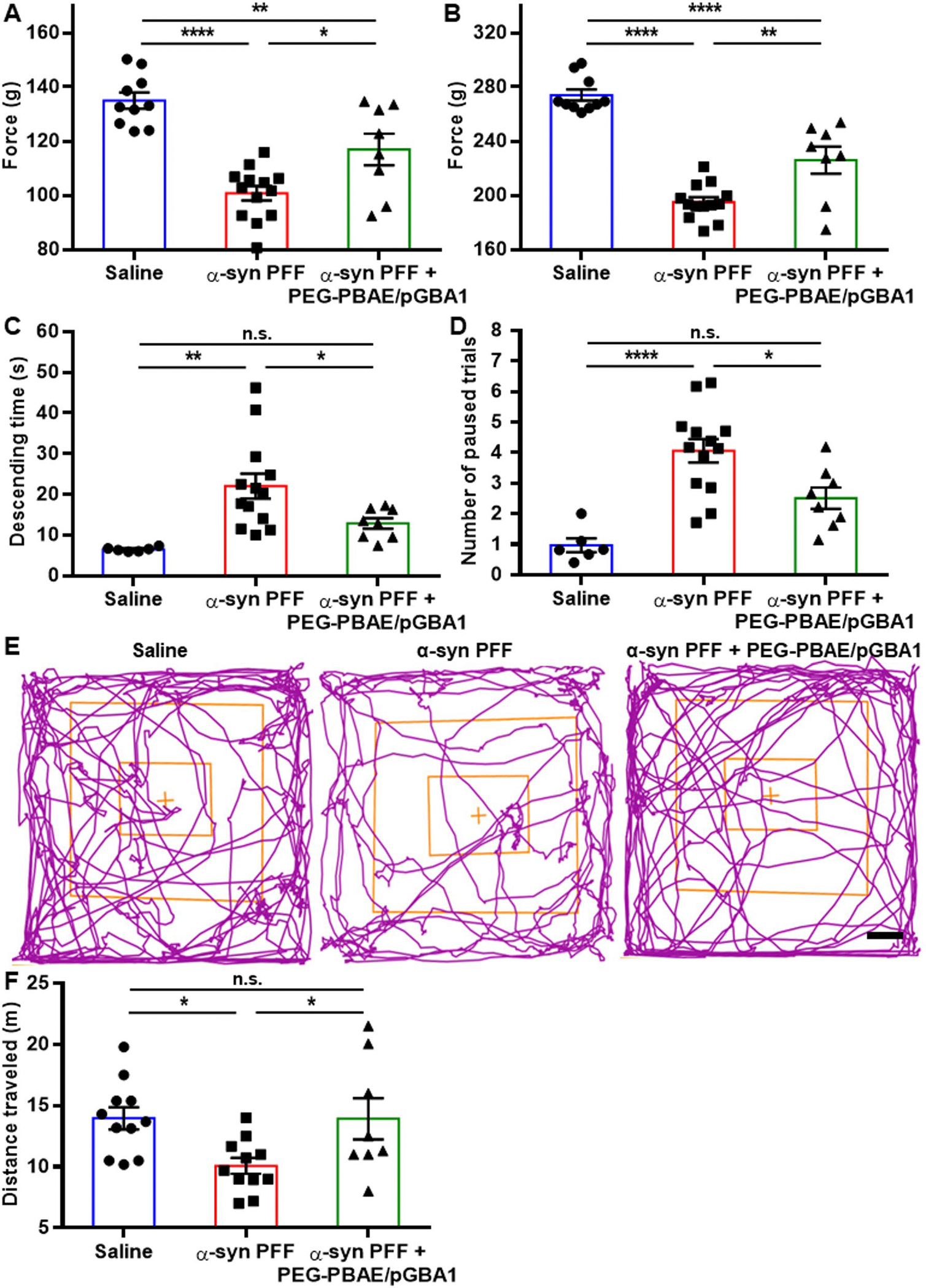
Overexpression of hGCase via SN-targeted CED of PEG-PBAE/pGBA1 restores neurobehavior deficits in an α-syn PFF-induced mouse model of PD. Neurobehavior functions of mice received saline or α-syn PFF with or without PEG-PBAE/pGBA1 treatments were tested at the 3-month time point. Quantification of maximum holding force using (**A**) forelimbs only or (**B**) fore- and hindlimbs measured in the grip strength test. (**C**) Quantification of descending time and (**D**) the number of paused trials measured in the pole test. (**E**) Representative tracking plot tracing mouse movements and (**F**) quantification of traveled distance measured in the open field test. Scale bar = 5 cm. n.s.: no significance, *p < 0.05, **p < 0.01, ****p < 0.0001 (one-way ANOVA followed by Tukey’s post-hoc test)

To assess the effects of PEG-PBAE/pGBA1 on motor deficits in the α-syn PFF-induced PD model, we conducted behavioral tests, including grip strength, pole, and open field tests, three months post-striatal saline or α-syn PFF injection. The grip strength test, which measures muscle strength [50] revealed a reduction in peak gripping forces in the α-syn PFF group, measuring 100.7 ± 0.7 and 195.0 ± 1.0 g for forelimbs only and fore- and hindlimbs, compared to 135.0 ± 0.9 and 274.5 ± 1.3 g in the saline group, respectively (Fig. 5A and B). PEG-PBAE/pGBA1 treatments significantly restored these forces to 116.9 ± 2.1 and 226.0 ± 3.5 g, respectively (Fig. 5A and B). In the pole test, which assesses motor coordination and agility [50], α-syn PFF group took significantly longer to descend (22.1 ± 0.9 s) compared to the saline group (6.5 ± 0.1 s) (Fig. 5C). PEG-PBAE/pGBA1 treatments significantly reduced the descending time to 12.9 ± 0.5 s (Fig. 5C). The number of paused trials during the descent in pole test, which reflects hesitation and akinesia, was also significantly greater in the α-syn PFF group (4.1 ± 0.1 trials) than saline group (1.0 ± 0.1 trials) (Fig. 5D), but PEG-PBAE/pGBA1 treatments significantly reduced the number of paused trials to 2.5 ± 0.1 (Fig. 5D). However, the time taken for the head to turn downward was significantly prolonged in the α-syn PFF group and was not recovered by PEG-PBAE/pGBA1 treatments (Figure S9). In the open field test, movement tracking revealed significant behavioral impairments in the α-syn PFF group, which were restored in the α-syn PFF + PEG-PBAE/pGBA1 group (Fig. 5E). The total traveled distance was significantly reduced in the α-syn PFF group (10.1 ± 0.2 m) compared to the saline group (13.9 ± 0.3 m) (Fig. 5F). PEG-PBAE/pGBA1 treatments restored the total traveled distance to 13.9 ± 0.6 m (Fig. 5F). Additional parameters, including mobile time, immobile time, center square entries, center square time, peripheral time, and the number of center square line crossings, did not show significant differences between the α-syn PFF group and the α-syn PFF + PEG-PBAE/pGBA1 group, but significant improvement was shown for peripheral entries (Figure S10).

### PEG-PBAE/pGBA1 treatments reduce neuroinflammation in the α-syn PFF-induced mouse model of PD

**Fig. 6:**
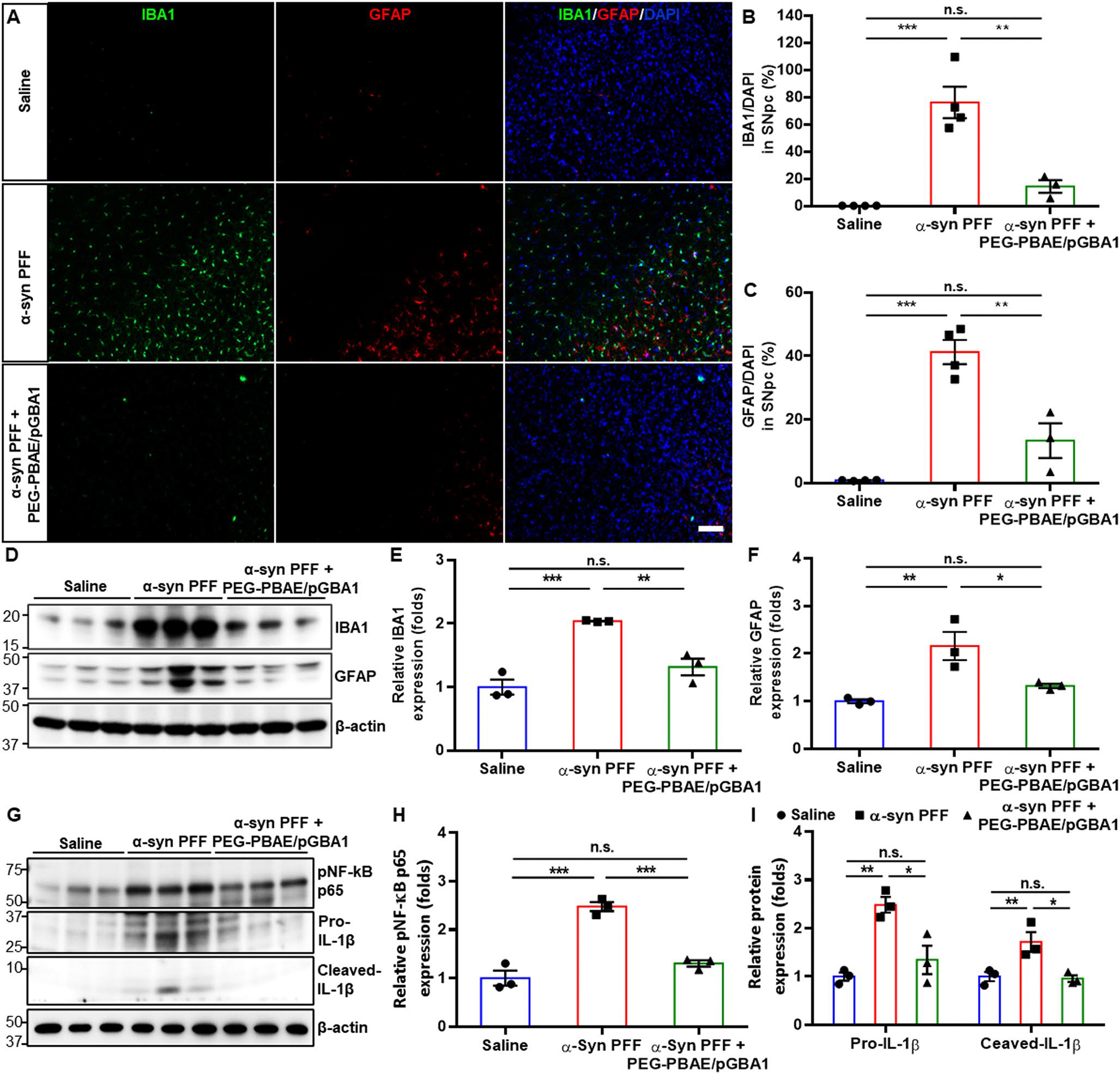
Overexpression of hGCase via SN-targeted CED of PEG-PBAE/pGBA1 reduces neuroinflammation in the SN in an α-syn PFF-induced mouse model of PD. (**A**) Representative confocal images of the SN in mice received saline or α-syn PFF with or without PEG-PBAE/pGBA1 treatments, as described in the timeline in Fig. 2A. Scale bar = 100 μm. Quantification of (**B**) IBA1-positive and (**C**) GFAP-positive staining relative to the DAPI-labeled nucleus in the SNpc, based on image panels in A. (**D**) Representative WB images showing levels of IBA1, GFAP, and β-actin in the VMBs of mice received saline or α-syn PFF with or without PEG-PBAE/pGBA1 treatments, as described in the timeline in Fig. 2A. Molecular weights in kDa are indicated on the left side of blot images. Quantification of relative (**E**) IBA1 or (**F**) GFAP levels normalized by β-actin, based on the WB data in D. (**G**) Representative WB images showing levels of pNF-κB p65, pro- and cleaved-IL-1β, and β-actin in the VMBs of mice received saline or α-syn PFF with or without PEG-PBAE/pGBA1 treatments, as described in the timeline in Fig. 2A. Molecular weights in kDa are indicated on the left side of blot images. Quantification of relative (**H**) pNF-κB p65 or (**I**) pro- and cleaved-IL-1β levels normalized by β-actin, based on the WB data in G. n.s.: no significance, *p < 0.05, **p < 0.01, ***p < 0.001 (one-way ANOVA followed by Tukey’s post-hoc test)

We assessed neuroinflammation in the treated brains by IHC using IBA1 and GFAP, markers for microglia and astrocytes, respectively [58, 59]. In the SN, immunoreactivity of IBA1 and GFAP was increased in the α-syn PFF group but was reduced by PEG-PBAE/pGBA1 treatments (Fig. 6A). Image-based quantification showed an increase in IBA1/DAPI (76.2 ± 5.8%) and GFAP/DAPI (41.2 ± 1.9%) in the α-syn PFF group compared to saline group (0.2 ± 0.01 and 0.8 ± 0.03%, respectively) (Fig. 6B and C). PEG-PBAE/pGBA1 treatments significantly brought the levels down to 14.4 ± 2.7 (IBA1/DAPI) and 13.3 ± 3.1% (GFAP/DAPI) (Fig. 6B and C). Additionally, WB analysis revealed significant increases in IBA1 and GFAP levels in the α-syn PFF group compared to saline group, but PEG-PBAE/pGBA1 treatments significantly reduced the levels towards the normal levels (Fig. 6D and E, and 6F). We also quantified the expression of inducers of neuroinflammation, including pNF-κB p65 and cleaved-IL-1β [60–62], via WB analysis. The levels of pNF-κB p65 and cleaved-IL-1β were significantly increased in the α-syn PFF group compared to saline group but were restored to the saline group levels by PEG-PBAE/pGBA1 treatments (Fig. 6G and H, and 6I). In the WB analysis with anti-IL-1β antibody, we also observed that the expression of pro-IL-1β, an inactive precursor of IL-1β, in the α-syn PFF group also significantly increased compared to saline group and was normalized by PEG-PBAE/pGBA1 treatments (Fig. 6I).

## Discussion

Our study provides compelling evidence that non-viral PEG-PBAE/pGBA1 therapy effectively inhibits neurodegeneration in an α-syn-PFF-induced mouse model of PD. PEG-PBAE NPs, due to their ability to retain colloidal stability in the brain and resist adhesive interactions with brain ECM [32, 33], enabled comprehensive coverage and overexpression of hGCase in the SN of both healthy and α-syn-PFF-induced PD mice following intracranial CED. Notably, PEG-PBAE/pGBA1 treatments significantly reduced the level of pS129 α-syn aggregation/accumulation in the SN and protected DA neurons in the SN and striatum comparable to the levels of the disease-free saline group. Additionally, significant improvements in motor functions were observed in the grip strength, pole, and open field tests, with no changes in non-motor (e.g., anxiety) parameters in the open field test. Further, PEG-PBAE/pGBA1 treatments markedly attenuated neuroinflammation which can exacerbate the neurodegenerative process of PD. These findings suggest that PEG-PBAE/pGBA1 may serve as a viable DMT that addresses multiple mechanisms of lysosomal dysfunction caused by GBA1 gene mutations or mutation-free reduction of GCase activity, offering more than simple symptomatic relief for a broad PD population. The ability of PEG-PBAE NPs to achieve widespread neuronal transgene expression also suggests the potential of the NP-mediated GBA1 gene therapy as a viable therapeutic strategy for other neurodegenerative disorders characterized by lysosomal dysfunction, beyond PD.

Remarkably, intracranially administered PEG-PBAE NPs enabled comprehensive human GBA1 transgene expression in TH-positive DA neurons in the SN. A recent study demonstrated that hGCase secreted from iPSC-derived neural precursor cells was taken up by nearby Gba1−/− neurons to restore their cellular GCase activity [63]. The finding suggests that functional hGCase produced by the cells successfully transfected with PEG-PBAE/pGBA1 can be shared with adjacent DA neurons with reduced GCase activity as a bystander effect. To this end, the ability of PEG-PBAE NPs to mediate widespread therapeutic transgene expression in SN, regardless of the specific type of transfected cells, renders this non-viral gene delivery platform well-positioned to provide comprehensive restoration of GCase activity and thus therapeutic benefits in PD.

In various preclinical models of PD, AAV-mediated GBA1 gene therapy has demonstrated promising therapeutic outcomes, including enhanced GCase activity, reduced α-syn pathology, and improved motor function [25–27]. Our previous work showed that intracranial AAV serotype 5 (AAV5)-based GBA1 gene therapy protected against 1-methyl-4-phenyl-1,2,3,6-tetrahydropyridine-induced DA neuron loss and improved motor function in GBA1^L444P/+^ mice [28]. The encouraging outcomes in these AAV-based preclinical studies led to a Phase I/IIa clinical trial investigating AAV9-based GBA1 gene therapy in PD patients with at least one pathogenic GBA1 mutation (NCT04127578). Due to the clinical successes of several AAV-based gene therapy products developed for rare genetic disorders, the platform will hold its position as the first choice for other gene therapy applications. As described above, however, AAV carries several inherent concerns, including safety and immunogenicity issues as well as prohibitive costs [29–31, 64], limiting its widespread use for highly prevalent diseases such as PD. To this end, our virus-free delivery approach may fill this gap to benefit a broader PD population.

Although the present study demonstrates encouraging outcomes, the reliance on the invasive intracranial CED to SN can cause deleterious effects, including infection, edema, and neuronal damage [65], particularly more so when repeated treatments are needed. Recently, we have demonstrated that PEG-PBAE NPs, due to their ability to retain colloidal stability in serum and stably circulate in the bloodstream, can be efficiently delivered to the brain parenchyma following less invasive systemic administration when coupled with focused ultrasound (FUS)-mediated blood-brain barrier (BBB) opening [38]. Importantly, magnetic resonance guidance can be used to open the BBB in specific target brain areas by FUS, thereby enabling precise targeted delivery of the long-circulating PEG-PBAE NPs to striatum or SN [66]. Notably, PEG-PBAE NPs can be engineered to carry various nucleic acid payloads, including pDNA, mRNA, and/or short nucleic acids such as single-guide RNA [38] for various gene therapy applications, including genome editing.

## Conclusion

In summary, our study highlights the therapeutic potential of the virus-free PEG-PBAE NP-mediated GBA1 gene therapy for treating a broad PD population. We provide evidence that our therapeutic strategy is capable of comprehensively tackling pathological events observed in human PD patients, including reduction of α-syn aggregation/accumulation, preservation of PD neurons, mitigation of neuroinflammation, and restoration of motor functions. While AAV remains a mainstay delivery platform for in vivo gene therapy applications, PEG-PBAE NPs hold a unique position to serve as an alternative, particularly for PD and other prevalent neurodegenerative disorders (e.g., Alzheimer’s disease), due to their ability to mediate widespread neuronal transgene expression in the brain regardless of administration route while addressing several well-appreciated limitations of virus-based gene therapy products.

## Supplementary Material

Supplementary Material

**Supplementary Information** The online version contains supplementary material available at https://doi.org/10.1007/s13346-025-01944-3.

## Figures and Tables

**Table 1:** Physicochemical properties of PEG-PBAE NPs

PEG-PBAE NP		Hydrodynamicdiameter^a^(nm)	PDI^a^	ζ-potential^b^(mV)

**pDNA payload**	**pLuc**	65.2 ± 1.2	0.19 ± 0.01	2.4 ± 0.1

	**pGBA1**	67.5 ± 1.6	0.21 ± 0.01	2.1 ± 0.2

a Hydrodynamic diameter (number mean) and polydispersity index (PDI) were measured by DLS in 10 mM NaCl at pH 7.4. Mean ± SEM (*n* = 4)

## Data Availability

All data associated with this study are available in the main text or are available through the corresponding author upon request.
