# Supplementary material for: Polymeric nanoparticle-mediated GBA1 gene therapy is neuroprotective in a preclinical model of Parkinson’s disease
Source: Drug Deliv Transl Res. 2025 Aug 20;16(3):894–910. doi: 10.1007/s13346-025-01944-3 (PMC12426995; doi:10.1007/s13346-025-01944-3)
Supplement: Supplementary file 1 — Supplementary Material 1 [file 13346_2025_1944_MOESM1_ESM.docx]

**Polymeric nanoparticle-mediated GBA1 gene therapy is neuroprotective in a preclinical model of Parkinson's disease.**

Mohit Kwatra^1,2,a^, Gijung Kwak^3,4,a^, Haolin Li^3,4,5^, Jung Soo Suk^3,4,5,6,*^, Han Seok Ko^1,2,*^

^1^Institute for Cell Engineering, School of Medicine, Johns Hopkins University, Baltimore, MD 21205, USA.

^2^Department of Neurology, School of Medicine, Johns Hopkins University, Baltimore, MD 21205, USA.

^3^Department of Neurosurgery, School of Medicine, University of Maryland, Baltimore, MD 21201, USA.

^4^Medicine Institute for Neuroscience Discovery (UM-MIND), School of Medicine, University of Maryland, Baltimore, MD 21201, USA.

^5^Department of Chemical and Biomolecular Engineering, School of Engineering, Johns Hopkins University, Baltimore, MD 21218, USA.

^6^Department of Neurosurgery, School of Medicine, Johns Hopkins University, Baltimore, MD 21205, USA.

^a^These authors contributed equally to this work.

Present address of Mohit Kwatra: Department of Ophthalmology, College of Medicine, Medical University of South Carolina (MUSC), Charleston, SC 29425, USA.

^*^Corresponding authors: Jung Soo Suk, Ph.D. (jsuk@som.umaryland.edu) and Han Seok Ko, Ph.D. (hko3@jhmi.edu)

**
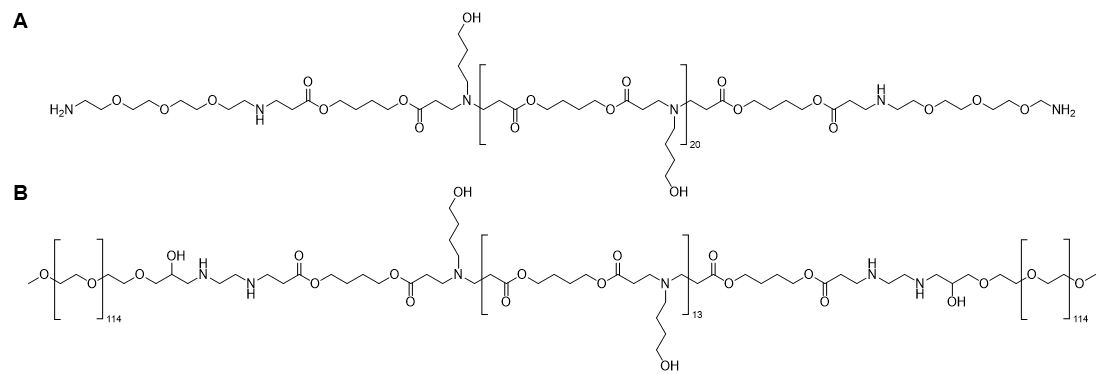
**

**Supporting Figure 1. Chemical structure of (A) PBAE and (B) PEG-PBAE.**

**
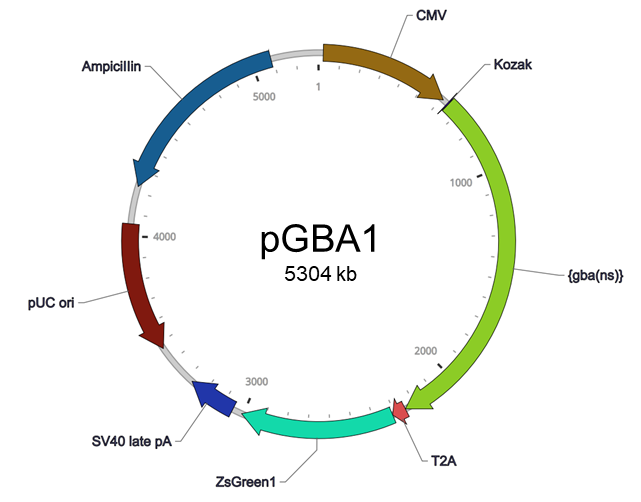
**

**Supporting Figure 2. Schematic map of pGBA1.**

**
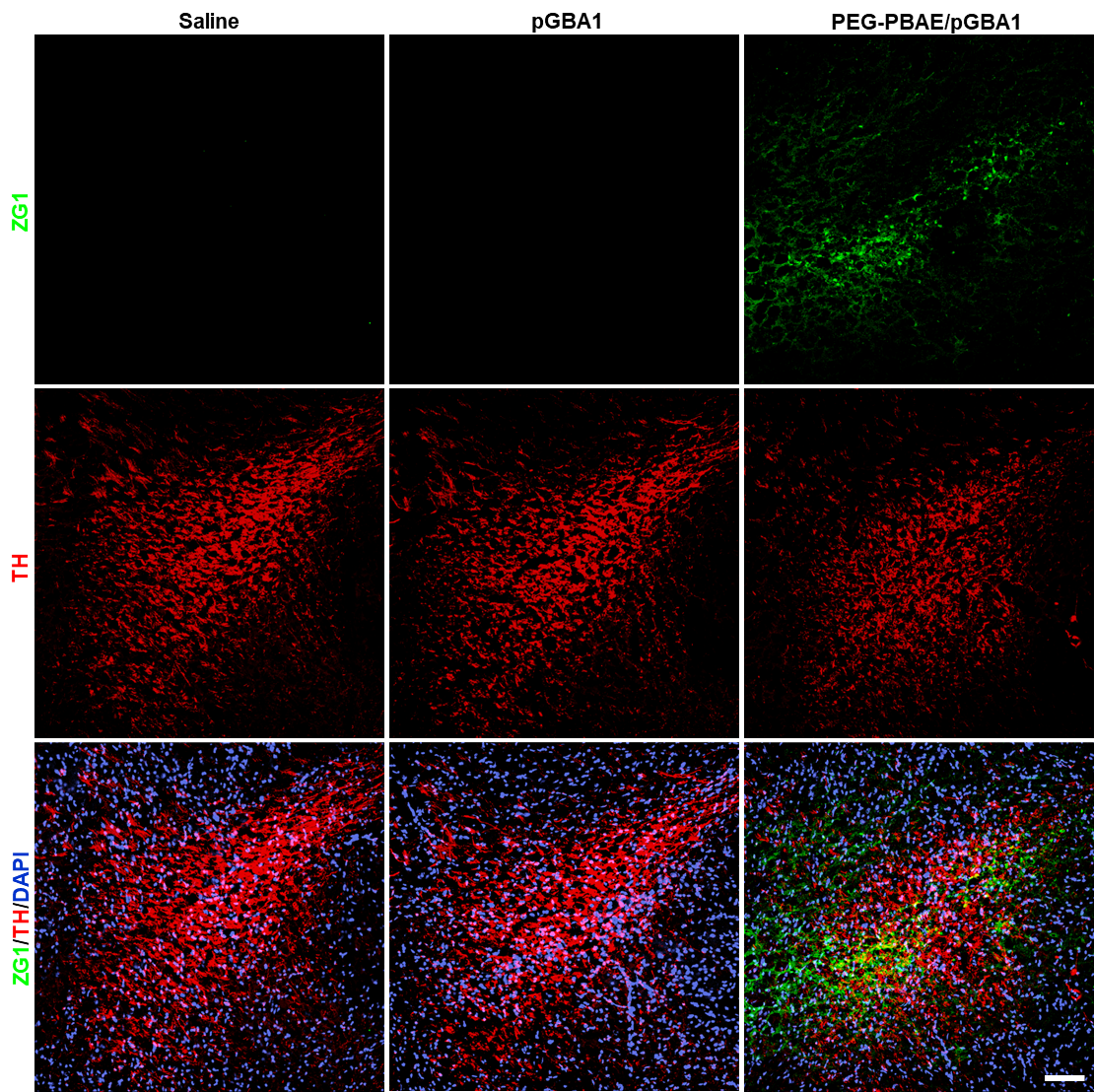
**

**Supporting Figure 3. PEG-PBAE/pGBA1, but not pGBA1, provides transgene expression in SN.** Representative confocal images of the SN area received saline, pGBA1, or PEG-PBAE/pGBA1 (2 μg pDNA). pGBA1 includes a reporter ZG1 encoding sequence. Scale bar = 100 μm.


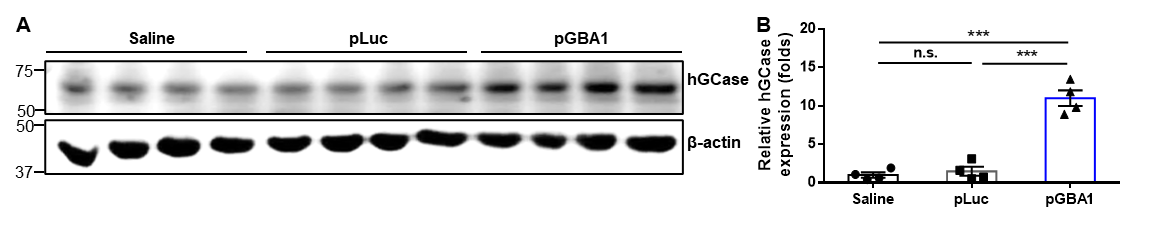


**Supporting Figure 4. Overexpression of hGCase by SN-targeted CED of PEG-PBAE/pGBA1 in healthy mouse brains. (A)** Representative WB images showing expression of hGCase and β-actin in the VMBs of mice received saline, PEG-PBAE/pLuc, or PEG-PBAE/pGBA1 (2 μg pDNA) via SN-targeted CED. **(B**) Quantification of relative hGCase expression normalized by β-actin expression, based on the WB data in A. n.s.: no significance, ****p* < 0.001 (one-way ANOVA followed by Tukey’s post-hoc test).

**
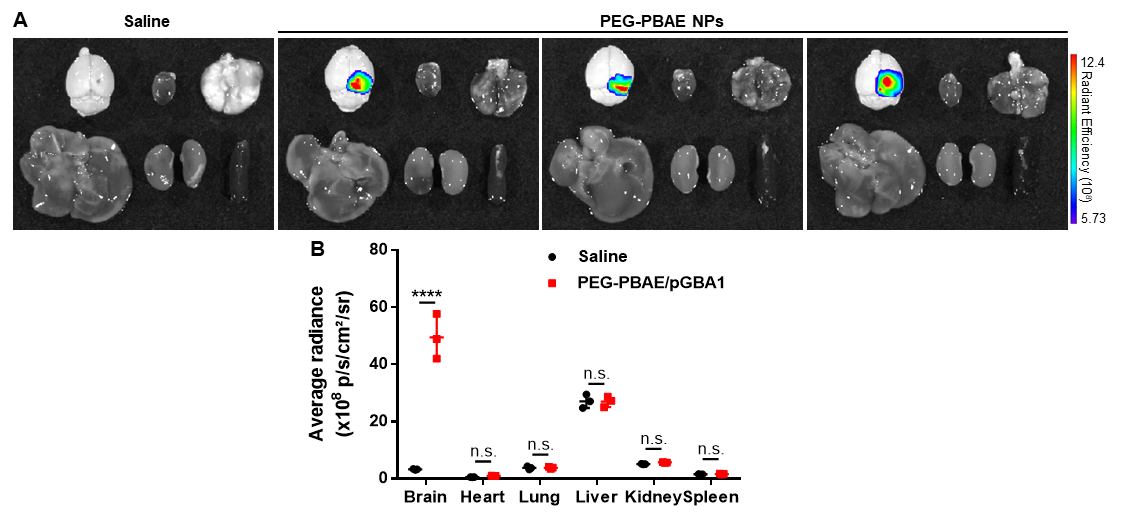
**

**Supporting Figure 5. PEG-PBAE NPs distribute only in the infusion site within the brain but are not found in the other peripheral organs. (A)** Representative IVIS images of the brain, heart, lung, liver, kidney, and spleen harvested from the animals received saline or Cy5-labeled PEG-PBAE/pGBA1 (2 μg pDNA) via SN-targeted CED. **(B)** Average radiance of each organ quantified in the IVIS images in A. n.s.: no significance, ****p < 0.0001 (two-way ANOVA followed by Bonferroni's multiple comparisons test).


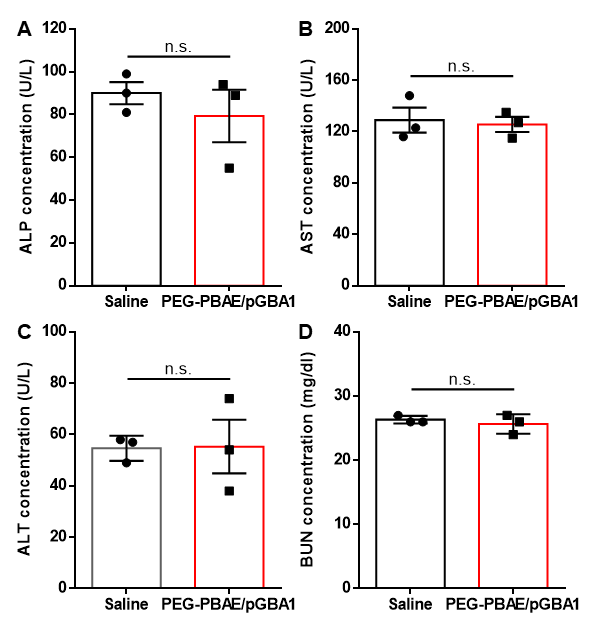


**Supporting Figure 6.** **Intracranially administrated PEG-PBAE/pGBA1 does not alter the metabolic function of the liver and kidney in healthy mice. (A)** Alkaline phosphatase (ALP), **(B)** aspartate transaminase (AST), **(C)** alanine transaminase (ALT), and **(D)** blood urea nitrogen (BUN) concentration in the serum harvested from the animals received saline or PEG-PBAE/pGBA1 (2 μg pDNA) via SN-targeted CED. n.s.: no significance (two-tailed unpaired t-test).

**Supporting Figure 7. Intracranially administrated PEG-PBAE/pGBA1 does not induce a humoral immune response as determined by the total IgG concentration in healthy mouse serum.** Total IgG concentration in the serum harvested from the animal’s received saline or PEG-PBAE/pGBA1 via SN-targeted CED. n.s.: no significance (two-tailed unpaired t-test).


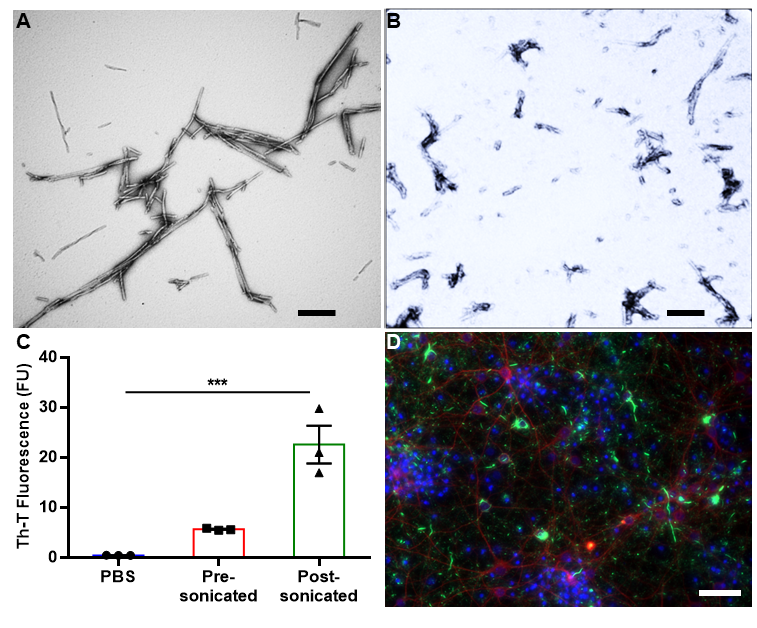


**Supporting Figure 8. Characterization of pre- and post-sonicated α-syn PFF and interaction with primary cortical neurons.** Transmission electron micrographs of **(A)** pre- and (B) post-sonicated α-syn PFF at a 2.5 mg/mL concentration. Scale bar = 500 nm. **(C)** Thioflavin-T (Th-T) fluorescence intensity of pre- and post-sonicated α-Syn PFF. **(D)** Representative IF image of primary cortical neurons treated with post-sonicated α-Syn PFF. Green: pS129 α-syn; red: neuronal MAP2; blue: nucleus. Scale bar = 50 μm. n.s.: no significance, ****p* < 0.001 (one-way ANOVA followed by Tukey’s post-hoc test).

**Supporting Figure 9. Quantitative parameters in pole test.** Head turn time measured in the pole test for mice received saline or α-syn PFF with or without PEG-PBAE/pGBA1 treatments, as outlined in the timeline in Figure 2A. n.s.: no significance, **p* < 0.05, ***p* < 0.01, ****p* < 0.001 (one-way ANOVA followed by Tukey’s post-hoc test).


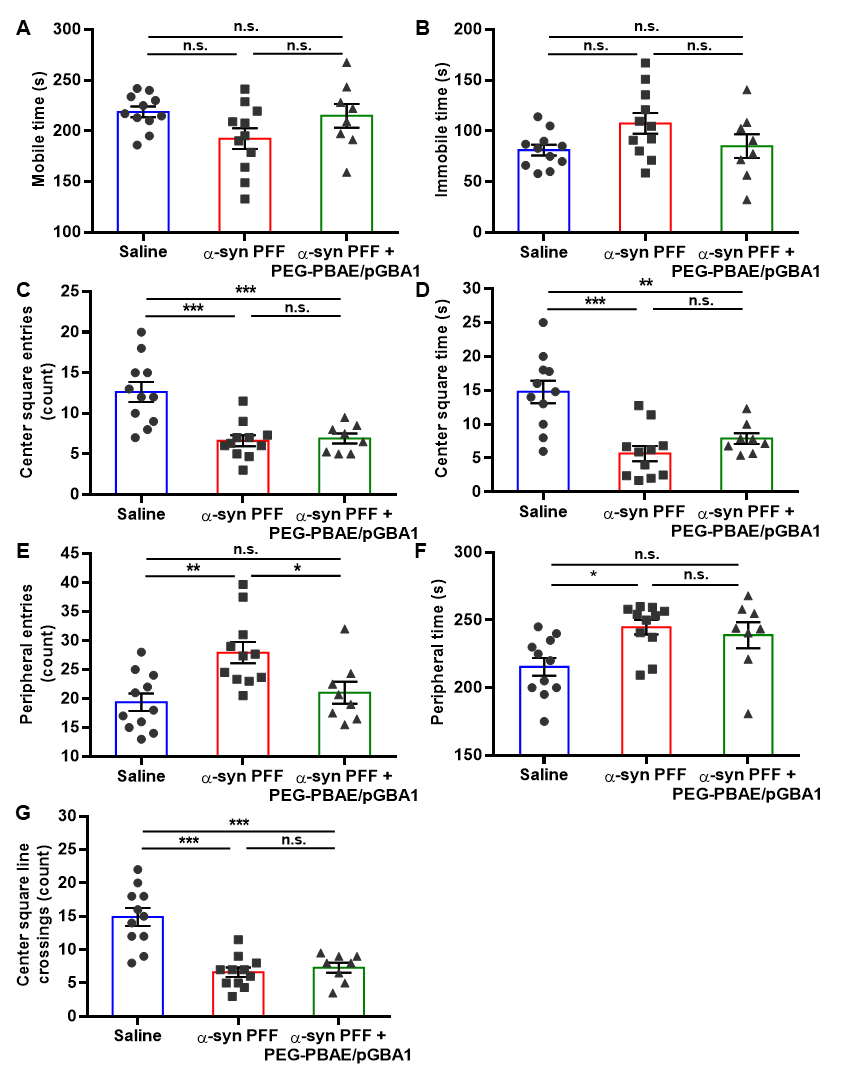


**Supporting Figure 10. Quantitative parameters in Open Field test. (A)** Mobile time, **(B)** immobile time, **(C)** center square entries, **(D)** center square time, **(E)** peripheral entries, **(F)** peripheral time, and **(G)** center square line crossings measured in Open Field test for mice received saline or α-syn PFF with or without PEG-PBAE/pGBA1 treatments, as outlined in the timeline in Figure 2A. n.s.: no significance, **p* < 0.05, ***p* < 0.01, ****p* < 0.001 (one-way ANOVA followed by Tukey’s post-hoc test).
